# Identifying risk factors for in-stent restenosis in symptomatic intracranial atherosclerotic stenosis: a systematic review and meta-analysis

**DOI:** 10.3389/fneur.2023.1170110

**Published:** 2023-07-14

**Authors:** Ning Wang, Yuning Lu, Lei Feng, Dongdong Lin, Yuhai Gao, Jiong Wu, Ming Wang, Shu Wan

**Affiliations:** ^1^Brain Center, Zhejiang Hospital, Hangzhou, China; ^2^The Second Clinical Medical College, Zhejiang Chinese Medical University, Hangzhou, China

**Keywords:** intracranial atherosclerotic stenosis, percutaneous transluminal angioplasty and stenting, in-stent restenosis, risk factors, meta-analysis

## Abstract

**Background:**

In-stent restenosis (ISR) is an adverse and notable event in the treatment of intracranial atherosclerotic stenosis (ICAS) with percutaneous transluminal angioplasty and stenting (PTAS). The incidence and contributing factors have not been fully defined. This study was performed to evaluate factors associated with ISR after PTAS.

**Data source:**

We identified studies on ISR after PTAS from an electronic search of articles in PubMed, Ovid MEDLINE, and the Cochrane Central Database (dated up to July 2022).

**Results:**

A total of 19 studies, including 452 cases of ISR after 2,047 PTAS, were included in the meta-analysis. The pooled incidence rate of in-stent restenosis was 22.08%. ISR was more likely to occur in patients with coronary artery disease (OR = 1.686; 95% CI: 1.242–2.288; *p* = 0.0008), dissection (OR = 6.293; 95% CI: 3.883–10.197; *p* < 0.0001), and higher residual stenosis (WMD = 3.227; 95% CI: 0.142–6.311; *p* = 0.0404). Patients treated with Wingspan stents had a significantly higher ISR rate than those treated with Enterprise stents (29.78% vs. 14.83%; *p* < 0.0001).

**Conclusions:**

The present study provides the current estimates of the robust effects of some risk factors for in-stent restenosis in intracranial atherosclerotic stenosis. The Enterprise stent had advantages compared with the Wingspan stent for ISR. The significant risk factors for ISR were coronary artery disease, dissection, and high residual stenosis. Local anesthesia was a suspected factor associated with ISR.

## Introduction

Intracranial atherosclerotic stenosis (ICAS) leads to a dramatic decline in cerebral perfusion and is the main cause of approximately 8%-10% of all ischemic strokes (1, 2). Current treatments for ICAS include medical and endovascular therapies, but rarely surgical therapy. Percutaneous transluminal angioplasty and stenting (PTAS) is considered a minimally invasive approach to reduce stroke recurrence in patients with symptomatic ICAS and has shown potential efficacy and acceptable periprocedural morbidity in initial studies (3–6). Stents commonly used in PTAS include self-expanding stents (SES) and balloon-expandable stents, each with its own advantages and disadvantages. Balloon-expandable stents have relatively rapid one-step exchange systems that do not need more complex exchange length guidewires than self-expanding stents (7, 8). In addition, with balloon-mounted stents (BMS), the lesion does not need to be navigated more than once, which may reduce the risk of embolic stroke and hemorrhagic complications (9–11). In-stent restenosis (ISR) is an adverse and notable event in PTAS, especially with balloon-mounted bare-metal stents, and has been shown to be reduced with drug-eluting stents (12, 13). It is significantly associated with long-term stroke recurrence in stent-treated patients. The incidence of ISR varies from 5% to 30% in present studies, and systematic research on risk factors for ISR is still lacking (14–17). To investigate the risk factors related to in-stent restenosis, we performed this meta-analysis.

## Materials and methods

### Search strategy

This study searched the following electronic databases for potentially relevant studies published up to July 2022: PubMed, Ovid MEDLINE, and the Cochrane Central Database. The keywords and medical subject headings (MeSH) used in the searches were “Arterial Disease, Intracranial” OR “Intracranial Arterial Disease” OR “Intracranial Arterial Disorders” OR “Arterial Disorder, Intracranial” OR “Arterial Disorders, Intracranial” OR “Intracranial Arterial Disorder” OR “Arterial Diseases, Intracranial” OR “Brain Diseases, Arterial” OR “Arterial Brain Disease” OR “Arterial Diseases, Brain” OR “Arterial Disease, Brain” OR “Brain Arterial Disease” OR “Brain Arterial Diseases” OR “Brain Disorders, Arterial” OR “Arterial Brain Disorder” OR “Arterial Brain Disorders” OR “Brain Disorder, Arterial” OR “Arterial Brain Diseases” AND “risk factor” OR “risk factors” AND “restenosis”. These words were combined using the Boolean operators OR and. The articles were limited to English as the only language of publication. In addition, the references listed in the identified articles were manually read to identify any additional eligible articles; a research assistant obtained and reviewed all potentially relevant articles.

Two authors independently analyzed the titles and abstracts of the identified articles. Inclusion criteria were as follows: (1) Full-length, peer-reviewed publications on stents for symptomatic ICAS in which the onset of restenosis was related to specific variables such as patient characteristics, stent technique, and other factors; (2) Cohort studies and single-arm studies were defined based on the study protocol; (3) Adequate data were presented to enable the computation of odds ratios (ORs) or weighted mean differences (WMDs) with 95% confidence intervals (CIs); (4) The median follow-up was at least 6 months; and (5) There were at least 10 patients per treatment group. Studies with any of the following characteristics were excluded: (1) Commentaries, reviews, protocols, letters, editorials, animal studies, or case reports; (2) Studies investigating the treatment strategy for complex cerebral artery stenosis; (3) Studies with imaging evaluation or treatment of ISR; and (4) Patients were treated without stent deployment. Disagreements in the evaluation of study inclusion were resolved by consensus between the two authors.

### Data extraction and quality assessment

Data were extracted from all eligible studies by the two authors with a structured data extraction form. The following characteristics were extracted from each study: name of the first author, year of publication, country, risk factors for ISR, number of patients in the ISR and control groups, and the number of patients with each potential risk factor for ISR. Any disagreement was resolved by discussion, and consensus was reached on all data. The definition of ISR was an angiographically verified >50% stenosis after stent deployment. The quality of the included cohort studies was assessed in conformity with the Newcastle–Ottawa Scale (NOS) (18), which is recommended by the Cochrane Collaboration as a bias assessment tool in observational studies. Single-arm studies were assessed using the Methodological Index for Nonrandomized Studies (MINORS) (19). Studies with a MINORS score of >10 or a NOS score of >5 were considered high-quality studies.

### Meta-analyses

This study complied with the PRISMA (Preferred Reporting Items for Systematic Reviews and Meta-Analyses) guidelines. Q-test statistics were used to qualitatively test for heterogeneity between studies, with significance set at *p* < 0.10 (20), and then tested by *I*^2^ statistics, with *I*^2^ >50% regarded as quantitative inconsistency. In the case of significant heterogeneity (*p* < 0.10 or *I*^2^ >50%), a random effects model was used to calculate pooled ORs or WMDs; otherwise, a fixed model was utilized (21). A forest plot was used to graphically summarize the meta-analysis of significant risk factors. All analyses were performed with the “meta” and “metafor” packages of the R statistical and computing software, version 4.1.2 (http://www.r-project.org/).

The possibility of publication bias was assessed by the Egger test and by framing a funnel plot of the effect size of each study relative to the standard error (Supplementary Figures 2, 3). A sensitivity analysis was performed to investigate the potential sources of heterogeneity (Supplementary Figure 4). Data on comparable factors, such as BMS and SES or local and general anesthesia, were extracted from studies with comparable results for pooled analysis; otherwise, subgroup analyses were performed. The following factors were analyzed with subgroup analyses: study type of the included studies, anesthesia type, dual antiplatelet time, and stent.

## Results

### Literature search and basic features of the included studies

**Figure 1:**
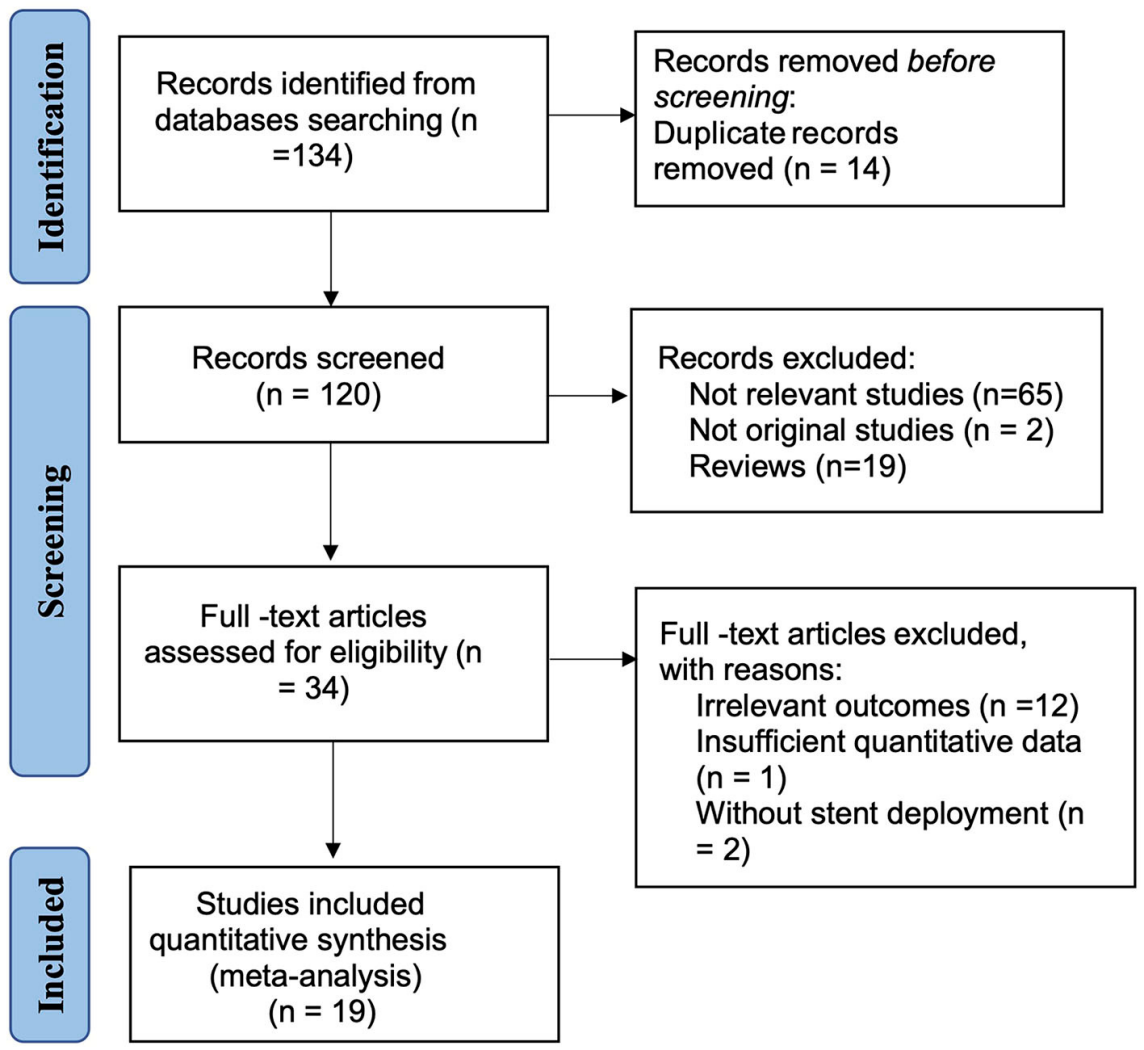
Flow diagram of the literature search.

A total of 136 references were initially evaluated; 19 studies were confirmed as eligible. These consisted of 9 cohort studies and 10 single arm studies, which included 452 cases of ISR after 2,047 PTAS, giving a cumulative incidence of ISR after PTAS of 22.08% (Supplementary Figure 1). In 17 studies, in-stent restenosis was defined as >50% stenosis at the time of angiographic follow-up, in one study as >70% stenosis, and as >20% increase in stenosis comparing to the residual post-procedural stenosis in the last study. The median follow-up for these studies was 12.6 months. All subjects were treated with aspirin and clopidogrel before the procedure and stayed on dual antiplatelet therapy for at least 3 months, or 6 months if necessary. The basic features of the included studies and participants are summarized in Table 1. The analysis of 17 potential risk factors for ISR was extracted from the included studies and is presented in Table 2. The PRISMA flow diagram of this analysis is presented in Figure 1.

**Table 1:** The detailed information on the basic characteristics of the 23 included studies and participants.

**Study**	**Year**	**Country**	**Design**	**Stent type**	**Restenosis**	**ISR symptoms**	**Total**	**Follow-up (mo)**	**Dual antiplatelet (mo)**	**Significant factors**
Levy et al. (15)	2007	America	single arm	Wingspan	25	4	80	5.9	3–6	Balloon diameter, posterior circulation
Turk et al. (22)	2008	America	cohort	Wingspan	29	9	93	7.3	3–6	Posterior circulation
Miao et al. (23)	2009	China	single arm	BMS	16	4	89	29	3	NR
Zhu et al. (24)	2010	China	single arm	BMS	18	3	61	7	6	Stent diameter, lesion length, diabetes
Al-Ali et al. (25)	2011	America	cohort	BMS and SES	30	NR	165	26	3–6	Lesion morphology
Li et al. (26)	2011	China	cohort	Wingspan	11	1	43	12.92	3	Balloon diameter equal to normal vessel
Yue et al. (27)	2011	China	cohort	BMS and SES	14	3	57	16.6	6	Stent type, residual stenosis
Jin et al. (28)	2013	China	cohort	BMS and SES	57	12	233	25.3	3–6	Gender, diabetes, smoking
Park et al. (29)	2013	Korea	cohort	BMS and SES	5	NR	19	6	6	NR
Shin et al. (30)	2013	Korea	single arm	Wingspan	17	3	69	18.9	3	Inflation speed
Feng et al. (31)	2015	China	single arm	Enterprise	3	0	44	25.6	1.5	NR
Wang et al. (32)	2016	China	single arm	Enterprise	6	5	45	6	3–6	Residual stenosis
Zhang et al. (33)	2016	China	cohort	BMS and SES	6	4	92	6–72	3	Residual stenosis
Ma et al. (34)	2018	China	single arm	BMS and SES	21	NR	76	12.5	3	Irregular medication intake
Guo et al. (35)	2021	China	cohort	BMS and SES	24	4	97	12.7	3	Stent length, hs-CRP, general anesthesia, stent type
Jia et al. (36)	2021	China	single arm	BMS and SES	8	NR	98	24	3–6	Diabetes, hypertension, coronary heart disease, age
Zhang et al. (37)	2021	China	cohort	Enterprise	62	NR	359	5.7	6	Residual stenosis, calcification, inflation pressure, nTICI
Haidegger et al. (38)	2021	Austria	single arm	Wingspan	38	7	115	11	3	Recurrent ischemic stroke
Yu et al. (39)	2021	China	single arm	BMS and SES	80	42	279	11	3	Residual stenosis, coronary heart disease

BMS, balloon-mounted stent; SES, self-expanding stent; NR, not reported.

**Table 2:** Detailed data on 15 potential risk factors and pooled results of meta-analyses.

**Potential risk**	**No. of studies**	**No. of patients**	**Pooled OR or WMD^d^**	**LL 95% CI**	**UL 95% CI**	***p*-value**	**Q-test (*p*)**	***I***
**Demographic variables**								
Age (y)	6	924	0.6245	−0.8723	2.1213	0.4135^a^	0.7604	0.00%
Gender (male)	8	1,044	0.8607	0.6143	1.2059	0.3833^a^	0.8066	0.00%
Smoking	6	894	1.1069	0.7883	1.5542	0.5577^a^	0.118	43.10%
Hypertension	9	1,386	1.2645	0.9065	1.7638	0.167^a^	0.6485	0.00%
Diabetes	9	1,386	1.412	0.9053	2.2023	0.1282^b^	0.0142	58.20%
Coronary artery disease	8	1,294	1.6858	1.2421	2.288	0.0008^a^	0.0951	42.50%
CRP	3	491	−0.3915	−7.8477	7.0684	0.918^b^	0.0035	82.30%
**Lesion variables**								
Posterior location	11	1,200	0.863	0.6445	1.1555	0.3225^a^	0.2498	20.30%
Stenosis grade (degree)	5	456	−0.4078	−2.5014	1.6858	0.7026^a^	0.6976	0.00%
Lesion length (mm)	5	456	0.4094	−1.517	2.3358	0.677^b^	0.0009	78.60%
Mori type A	3	442	0.5673	0.1005	3.2016	0.5208^b^	0.0073	79.70%
**Procedure-related variables**								
Residual stenosis (degree)	6	644	3.2268	0.1418	6.3117	0.0404^b^	< 0.0001	84.90%
Residual stenosis>30%	2	423	14.1472	1.4195	140.99	0.0239^b^	0.0042	87.80%
General anesthesia	3	441	0.6148	0.1952	1.9367	0.406^b^	0.0319	71.00%
Dissection	2	524	6.2926	3.8833	10.1968	< 0.0001^a^	0.0505	73.80%
Stent type	8	953	1.0309	0.8462	1.2559	0.7625^a^	0.1253	38.20%

CI, confidence interval; LL, lower limit; UL, upper limit; WMD, weighted mean difference; OR, odds ratio.

^a^Fixed-effects model was framed.

^b^Random-effects model was framed.

^c^I^2^ statistic was defined as the proportion of heterogeneity not due to chance or random error.

^d^Weighted mean difference (WMD) for continuous variables and odds ratio (OR) for bilateral variables.

### Methodological quality assessment

The outcome of the quality assessment of the included studies was as follows: within the single arm studies, four studies received a score of 14, four studies received a score of 12, and two studies received a score of 10; within the cohort studies, five studies received a score of 8, three studies received a score of 7, one study received a score of 6, and one study received a score of 5. Detailed information on the quality assessment is shown in Supplementary Tables 1, 2.

### Pooled analyses of risk factors

A meta-analysis of individual relative results indicated that various risk factors were associated with the development of ISR after PTAS (Table 2). The comprehensive OR ranged from 0.567 to 14.147. Significant heterogeneity between studies was observed for diabetes, lesion length, Mori type, residual stenosis > 30%, and CRP.

Regarding procedure-related variables, ISR was more likely to occur in patients with coronary artery disease (OR = 1.686; 95% CI: 1.242–2.288; *p* = 0.0008), dissection (OR = 6.293; 95% CI: 3.883–10.197; *p* < 0.0001), residual stenosis > 30% (OR = 14.147; 95% CI: 1.419–140.99; *p* = 0.0239) and higher residual stenosis (WMD = 3.227; 95% CI: 0.142–6.311; *p* = 0.0404) (Supplementary Figures 5–8).

Overall, ISR was not associated with gender, age, smoking, hypertension, diabetes, posterior location, degree of stenosis, lesion length, or stent type, which was divided into the self-expanding stent and the balloon-mounted stent (all *p* >0.05). All analysis outcomes are shown in Table 2.

### Subgroup analyses and heterogeneity

Subgroup analyses were performed to further investigate the risk factors for ISR. Different choices of anesthesia resulted in significantly different in-stent restenosis rates, with local anesthesia having the highest rate of ISR (18.4% vs. 25.8% vs. 33.0%; *p* = 0.0088) (Table 3). Patients treated with Wingspan stents were more prone to ISR than those treated with Enterprise stents (29.78% vs. 14.83%; *p* < 0.0001) (Supplementary Figures 9, 10). Study type and DAPT duration were not found to be associated with ISR (Table 3).

**Table 3:** Subgroup analysis of ISR patients.

	**Subgroup**	**Proportion**	**LL 95% CI**	**UL 95% CI**	***p*-value**
Design	Cohort	0.2018	0.144	0.2596	0.6954
	Single arm	0.2189	0.1557	0.2821	
Antiplatelet	Dual antiplatelet < 6M	0.2055	0.1557	0.2553	0.5877
	Dual antiplatelet ≥6M	0.2299	0.157	0.3029	
Anesthesia	Local anesthesia	0.3304	0.2445	0.4164	0.0088
	Local and general anesthesia	0.2584	0.2119	0.305	
	General anesthesia	0.1838	0.1325	0.235	
Stent type	Wingspan	0.2978	0.2531	0.3425	< 0.0001
	Enterprise	0.1483	0.1156	0.181	

CI, confidence interval; LL, lower limit; UL, upper limit.

Significant heterogeneity between effect estimates was found for the following variables: diabetes, CRP, lesion length, Mori type A, residual stenosis, residual stenosis >30%, general anesthesia, and dissection. Mild heterogeneity between effect estimates was observed for age, gender, hypertension, smoking, coronary artery disease, posterior location, degree of stenosis, and stent type.

## Discussion

ISR is an important post-procedural complication of PTAS. The present meta-analysis revealed that 22.18% of symptomatic ICAS patients may suffer from ISR after stent deployment. Risk factors for the development of ISR were identified as the Wingspan stent, coronary artery disease, dissection, and high residual stenosis. In addition, patients who received local anesthesia were more likely to develop ISR. ISR was not associated with gender, age, smoking, other morbidities, or other lesion characteristics.

Patients who were implanted with a Wingspan stent were more likely to develop in-stent restenosis than those treated with Enterprise. The ISR rate for the Enterprise procedure was found to be 14.83% compared to 29.78% for Wingspan. The Enterprise stent is a self-expanding, closed-cell stent that was originally designed for coiling assistance of wide neck intracranial aneurysms (40). This stent has been shown to perform better than the Wingspan stent in complex intracranial atherosclerotic stenoses due to its high flexibility, special carrier system structure, decreased radial force, and capability to reduce the risk of damage to the arteries and prevent elastic recoil and in-stent restenosis (31, 41). In addition, Zsolt et al. reported satisfactory ISR rates with the Enterprise stent (24.7% restenosis at 6 months follow-up) compared to the Wingspan stent (42). On the other hand, Xu et al. found that the ISR rate was significantly higher in the balloon-mounted stent group than in the Wingspan stent group (35), which was not supported by this meta-analysis. The present study revealed that patients treated with BMS had a similar ISR rate than those implanted with Wingspan. New neuro-interventional devices have been developed in recent years. For example, the drug-eluting stent was shown to have an ISR rate of 9.5% in a recent clinical trial (43), which is significantly lower than that of the present stent. Therefore, endovascular treatment of intracranial atherosclerotic stenosis will become increasingly accurate and effective with technological development.

This study found that ISR was significantly more common in patients with coronary artery disease (CAD). In fact, CAD shares the same pathology as ICAS, which is atherosclerosis of the vascular walls. The instability or rupture of atherosclerotic plaques can lead to both cardiovascular and cerebrovascular events (44, 45). Essentially, atherosclerosis appears to be an inflammatory disease, and inflammation plays an important role in the progression of atherosclerosis (46). The severity of atherosclerosis may partly reflect the level of inflammatory activity in the systemic vessel walls. ICAS patients with CAD may have more severe atherosclerosis in the systemic vessels, and the plaques may be prone to instability and rupture, which means a more vibrant systemic inflammatory response in the vessel walls and a greater opportunity for ISR. CRP is a representative inflammatory biomarker mainly produced by hepatocytes and has been suggested to be a strong predictor of intracranial ISR in two Chinese studies (35, 39). High CRP levels are a predictor of asymmetric progression of stenotic tissue because of the differential distribution of shear stress and the effect on neointimal tissue shape mediated by the inflammatory process (47). However, the association between CRP levels and ISR was not detected in Melanie et al. (38), which may be explained by the relatively old study population, as CRP increases with age and comorbidities (48). Therefore, the value of CRP as a predictive marker for ISR after stenting may be limited.

Dissection was another significant risk factor for ISR. Previous studies evaluated the association between dissection and ISR (25, 37), and concluded that dissection was associated with ISR. The presence of dissection during intervention indicates damage to the endarterium of the lesioned vessel, which may induce intimal hyperplasia and the inflammatory cascade. The exact pathophysiological mechanism requires further investigation. Therefore, a small balloon should be utilized for predilation, and the one utilized for dilatation should be selected with 80% of the diameter of the adjacent normal artery to avoid dissection of the atherosclerotic plaque (37).

High residual stenosis is also one of the risk factors for ISR. Yue et al. (49) and Zhang et al. (37) revealed that patients with residual stenosis > 30% were more likely to develop ISR than those with residual stenosis < 30%. In addition, eight other studies defined residual stenosis as a continuous variable, which summarized that patients in the restenosis group had a significantly higher stenosis rate immediately after the procedure. However, there were few detailed illustrations to explain the relationship between residual stenosis and ISR. Yue et al. suggested that higher residual stenosis might induce atherosclerotic plaques to protrude into the remodeled vessel. Some studies found the situation to be theoretically true. In our group, we thought it might be related to hemodynamics. For example, different residual stenosis rates resulted in different blood flow velocities and turbulence on either side of the stenosis.

The subgroup analysis other than the pooled analysis showed that ISR occurred more frequently in patients who underwent surgery with local anesthesia. Xu Guo et al. recently determined that local anesthesia was significantly associated with ISR (35), but Zhu et al. and Ying et al. did not reach this conclusion (24, 39). The management of anesthesia in the endovascular treatment of non-acute stroke patients with ICAS has been little discussed in the recent literature. Local anesthesia is easy to achieve during the surgical procedure, with the advantages of lower cost, less time consumption, and earlier detection of patient deterioration (24); however, it is hard for patients to keep still during the entire procedure. On the other hand, general anesthesia could minimize patient activity during surgery and allow for substantial submaximal inflation to be performed to reduce complications of technical surgery, such as iatrogenic perforation or dissection.

This study showed no association between ISR and age. Turk et al. (22) reported that ISR was more common in younger patients, with a cutoff age of 55 years. The authors hypothesized that the lesions in younger patients displayed more inflammatory arteriopathy than those with primary atherosclerosis. This study identified age as a continuous variable to be analyzed, and a negative correlation was found between the ISR and age. Different types of stents were also found not to be associated with ISR. Further studies are needed to identify whether younger patients or lesions with self-expanding stents have higher restenosis rates and physiopathological mechanisms.

The present study had several limitations that need to be discussed. First, only study-level data rather than raw data were extracted from the published literature, and the sample size in 80% of the series was < 100 patients. The target population of the studies varied with the inclusion criteria, resulting in limited generalization of population features such as distribution of lesion location, preprocedural stenosis grade, and proportion of stent type. Second, all included studies were nonrandomized observational studies, and specific biases were unavoidable. Third, the variables extracted from the studies were limited for the meta-analysis design. The complicated lesion morphology, balloon diameter of 80% of the normal vessel, stent length, inflation speed, irregular medication intake, calcifications, inflation pressure, and ulcerations, which may lead to ISR, were rarely mentioned in previous studies. This study also has its strengths, such as the comprehensive literature search, the careful evaluation of methodological quality, and the assessment of heterogeneity. In this respect, the level of evidence in this study was higher than that of some of the individual studies (50). The present results will give neurointerventionists suggestions on how to prevent ISR after PTAS and highlight the need for further studies on ISR after PTAS.

## Conclusions

The present study provides the current estimates of the robust effects of some risk factors for in-stent restenosis in intracranial atherosclerotic stenosis. The Enterprise stent had advantages compared to the Wingspan stent for ISR. The significant risk factors for ISR were coronary artery disease, dissection, and high residual stenosis. Local anesthesia was a suspected factor associated with ISR. Further studies should be conducted on patients undergoing PTAS for different inciting conditions to elucidate the underlying mechanism of ISR.

## Data availability statement

The original contributions presented in the study are included in the article/Supplementary material, further inquiries can be directed to the corresponding author.

## Author contributions

NW wrote the manuscript. NW and YG performed the statistical analyses. NW, YL, and LF gathered the data and responsible for the integrity of the extracted data. SW, JW, and MW designed and coordinated the study. DL contributed to the analysis and interpretation of the data. All authors contributed to the article and approved the submitted version.

## Supplementary material

Supplementary Figures 1–4 The cumulative incidence of ISR after PTAS along with a funnel plot, Egger test, and sensitivity analysis of the extracted data.

Supplementary Figures 5–8 Possible risk factors for ISR.

Supplementary Figures 9–10 The result of the subgroup analysis.

Supplementary Tables 1, 2 Detailed quality assessment information was provided.
