# Supplementary figures and images for: Identifying risk factors for in-stent restenosis in symptomatic intracranial atherosclerotic stenosis: a systematic review and meta-analysis
Source: Front Neurol. 2023 Jul 14;14:1170110. doi: 10.3389/fneur.2023.1170110 (PMC10375724; doi:10.3389/fneur.2023.1170110)

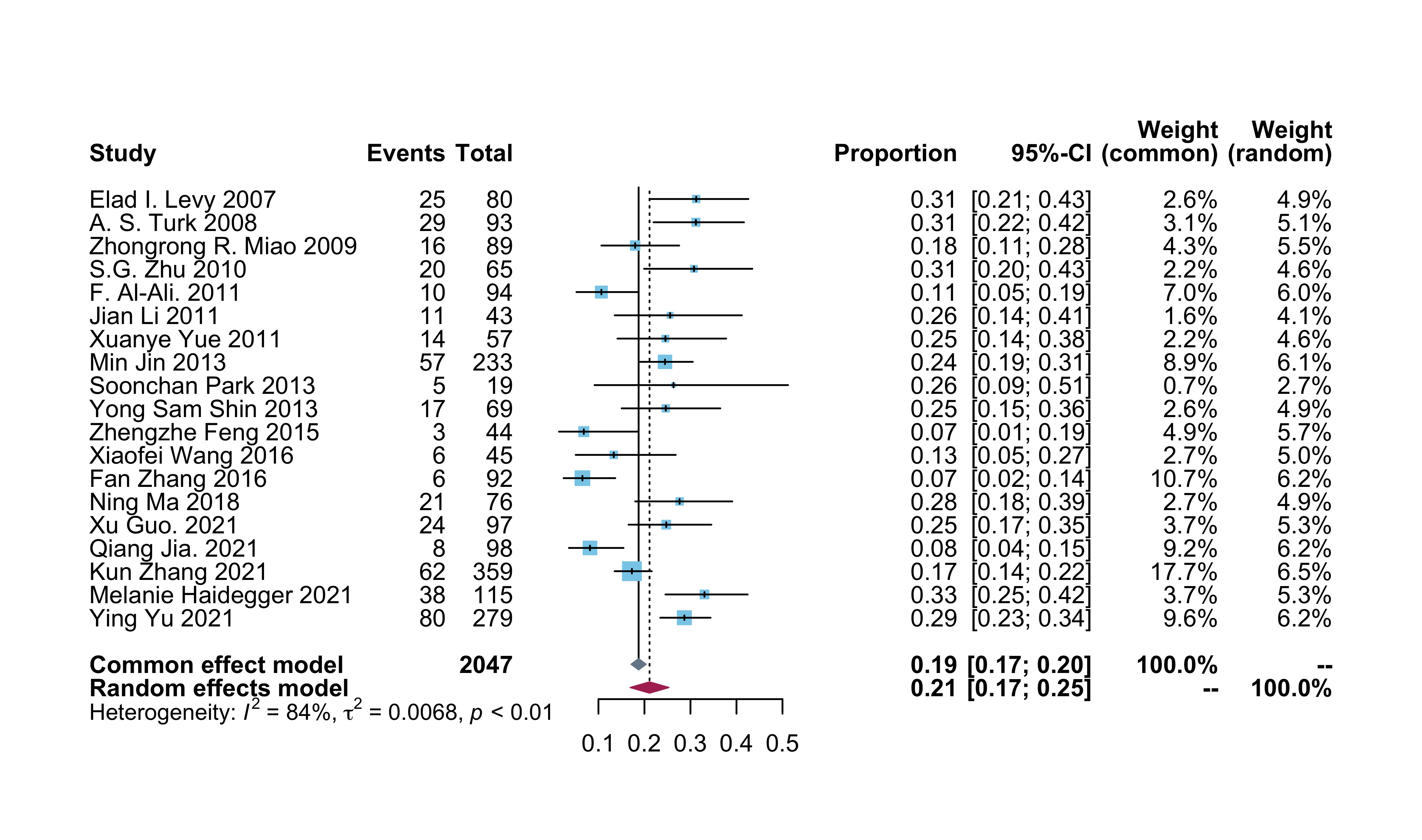

Supplement: Supplementary file 1 [file Image_1.JPEG]

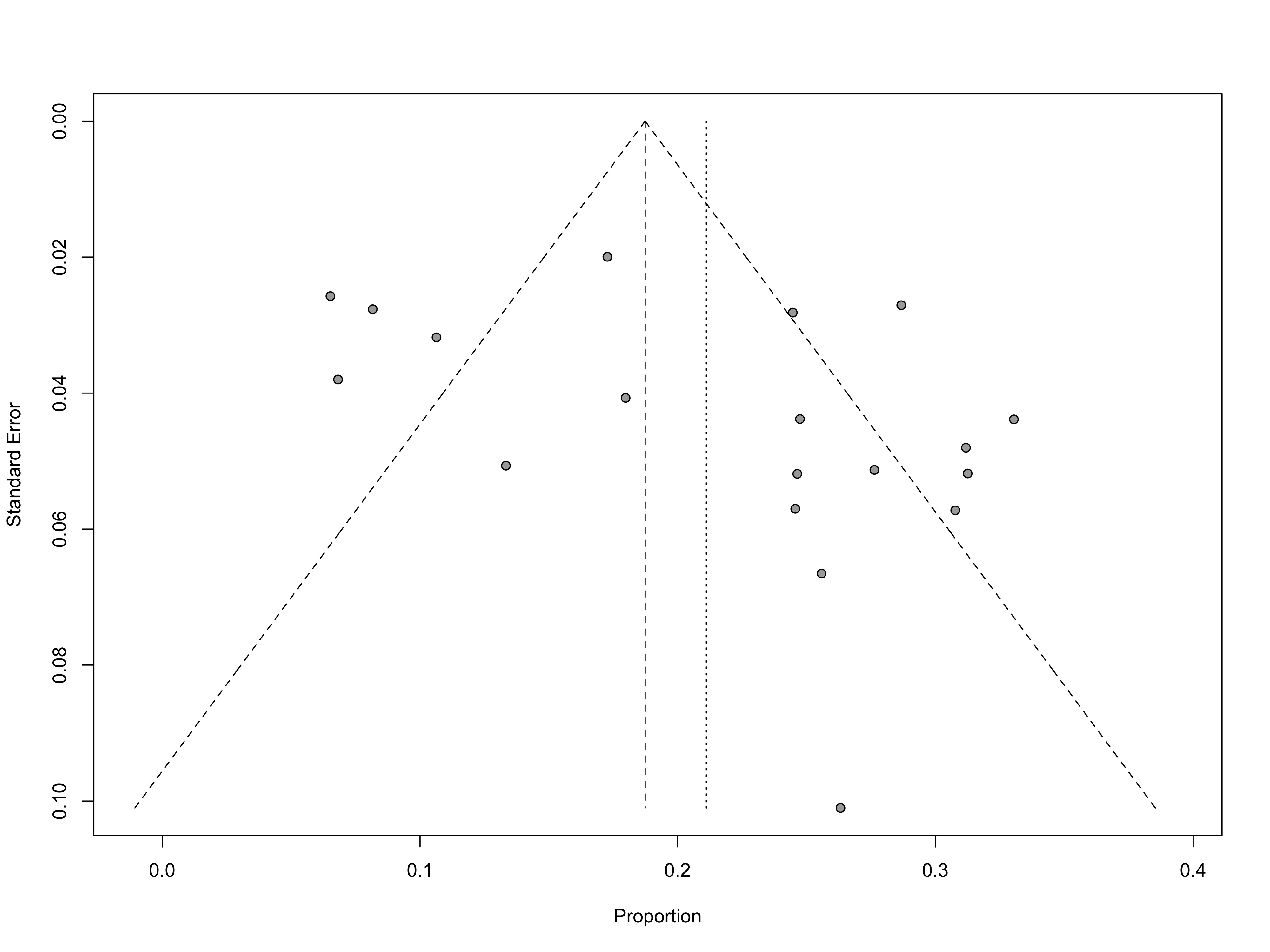

Supplement: Supplementary file 2 [file Image_2.JPEG]

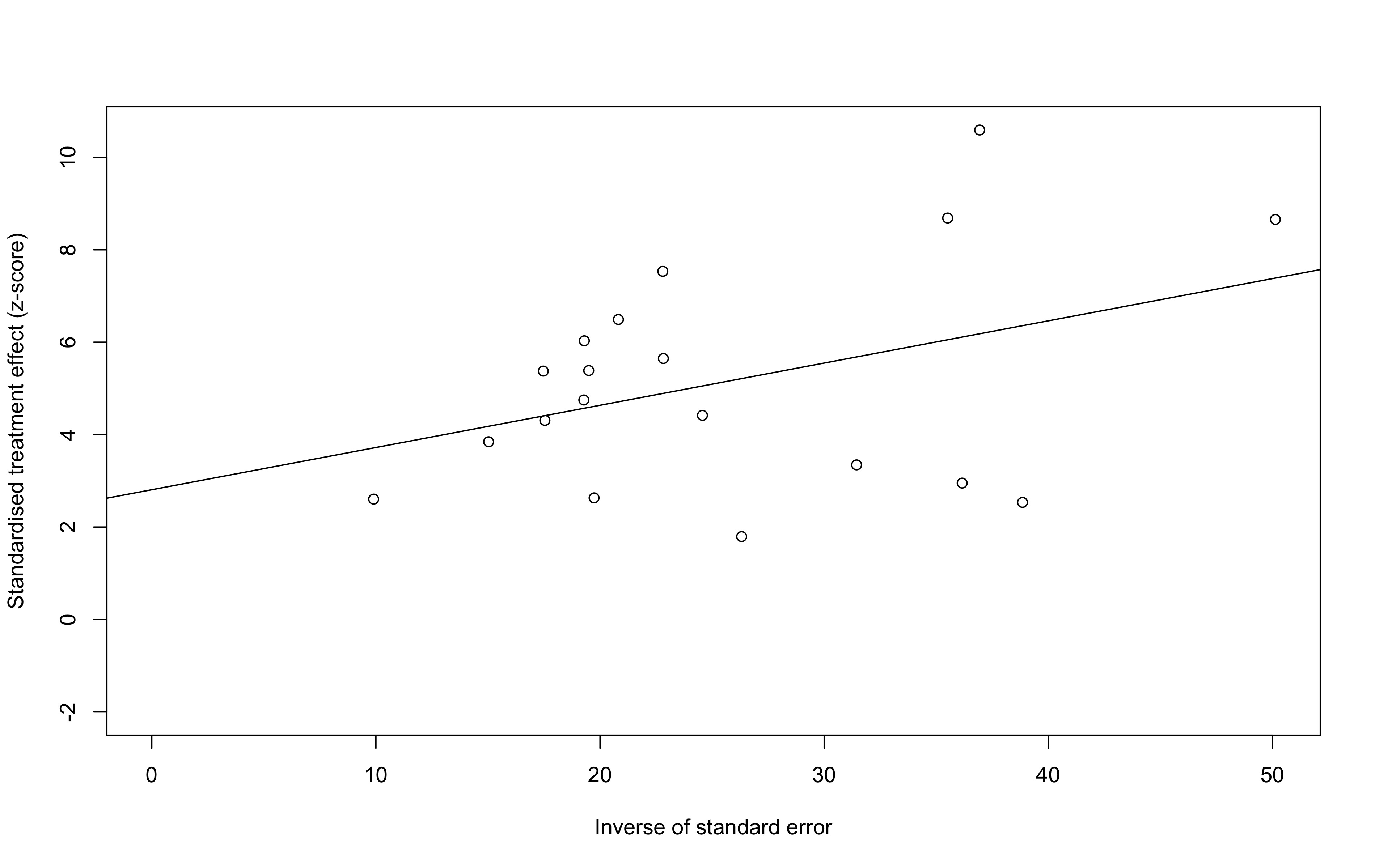

Supplement: Supplementary file 3 [file Image_3.JPEG]

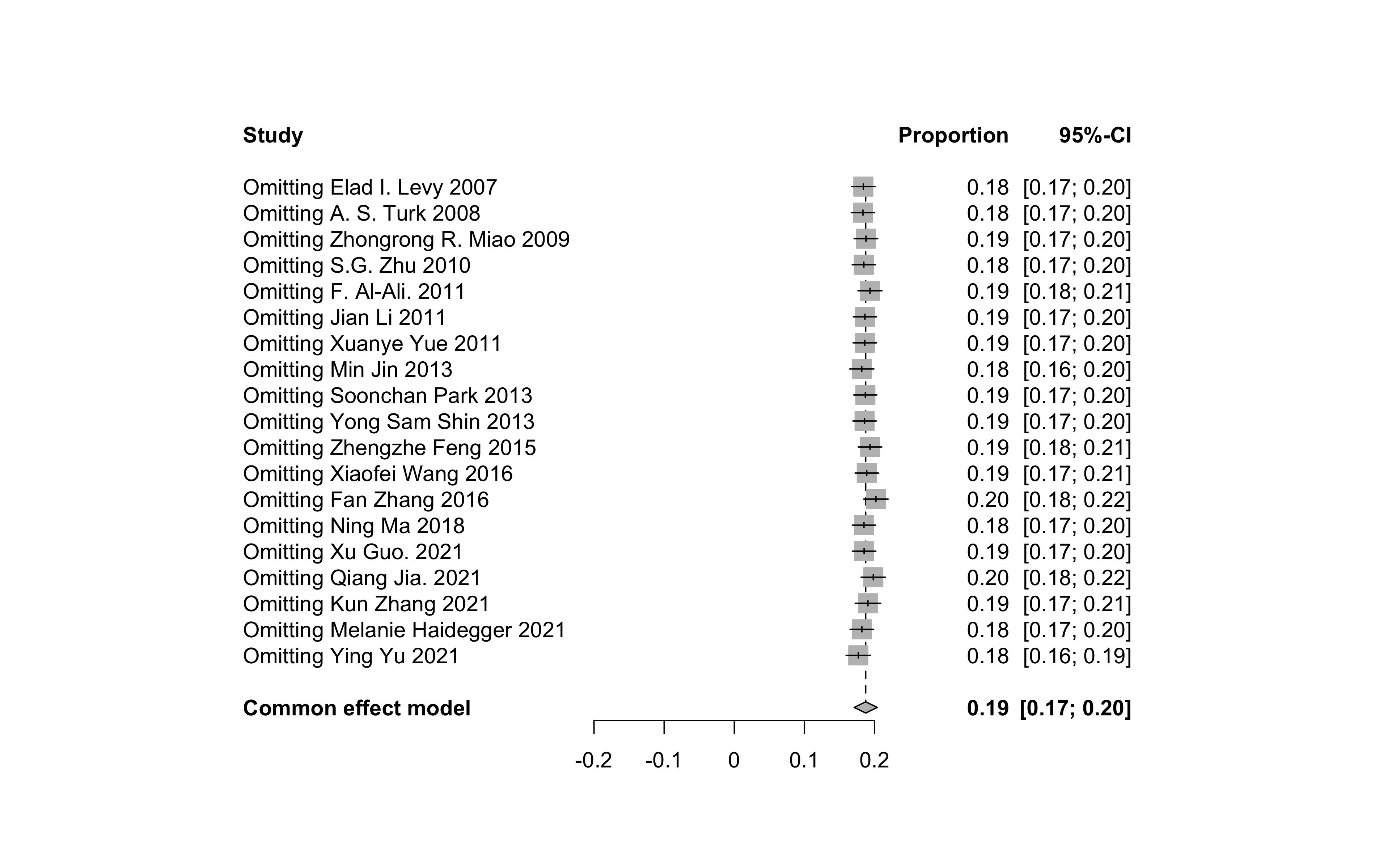

Supplement: Supplementary Figures 1–4 — The cumulative incidence of ISR after PTAS along with a funnel plot, Egger test, and sensitivity analysis of the extracted data. [file Image_4.JPEG]

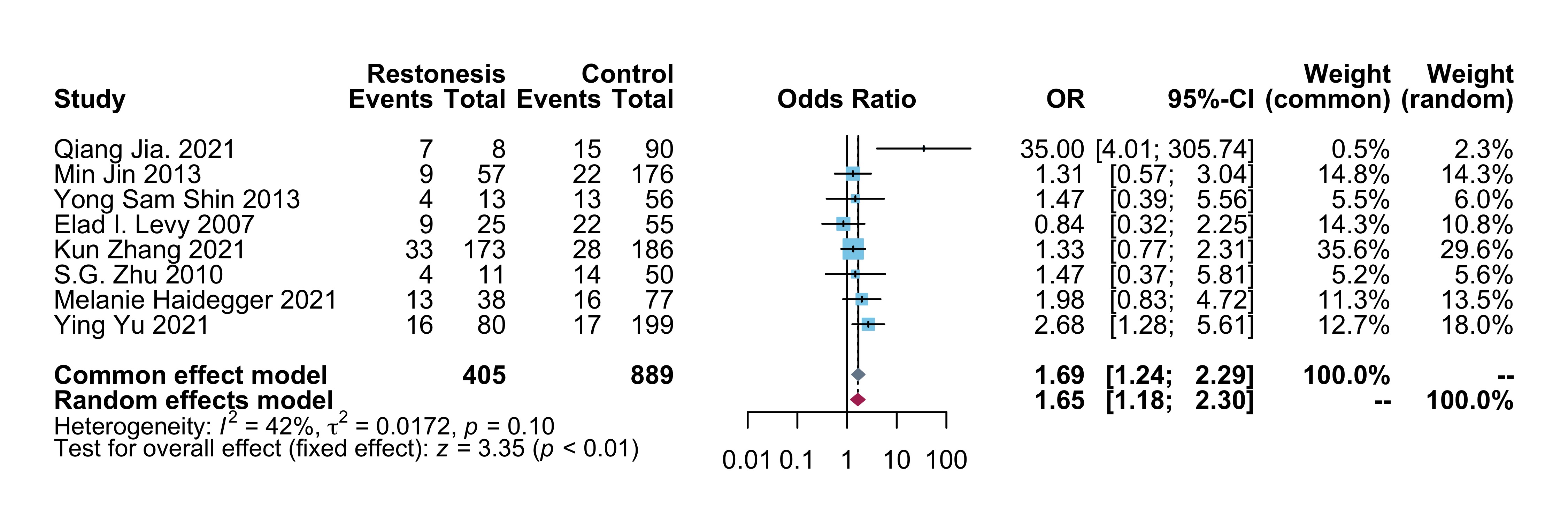

Supplement: Supplementary file 5 [file Image_5.JPEG]

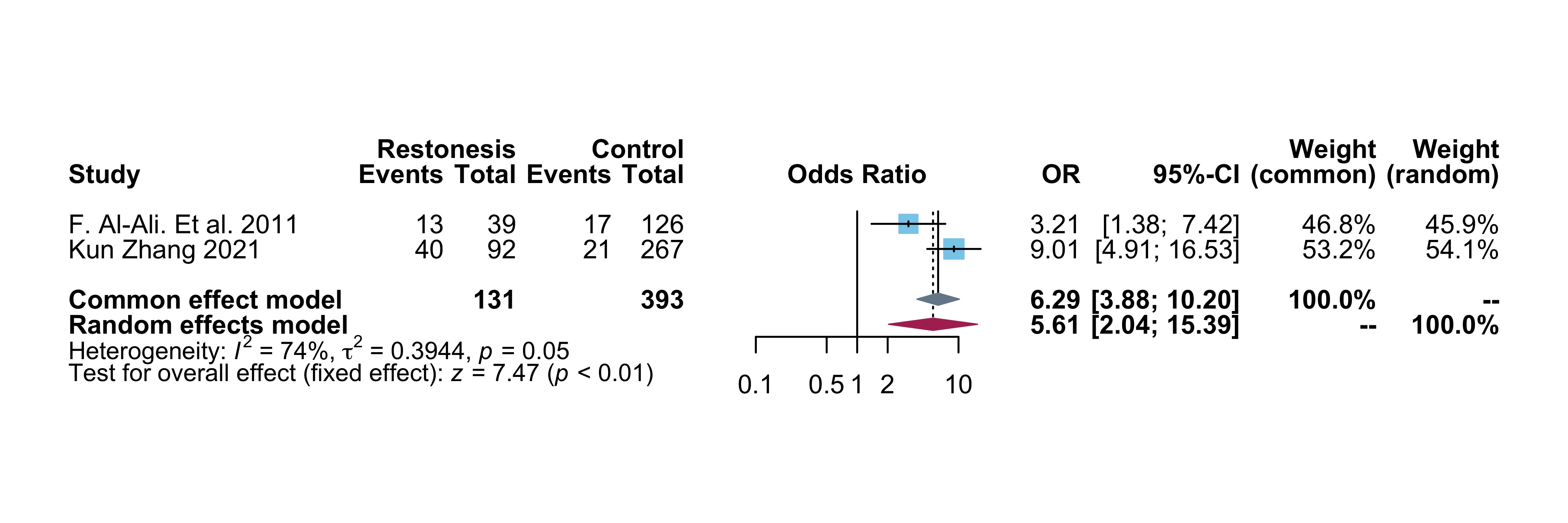

Supplement: Supplementary file 6 [file Image_6.JPEG]

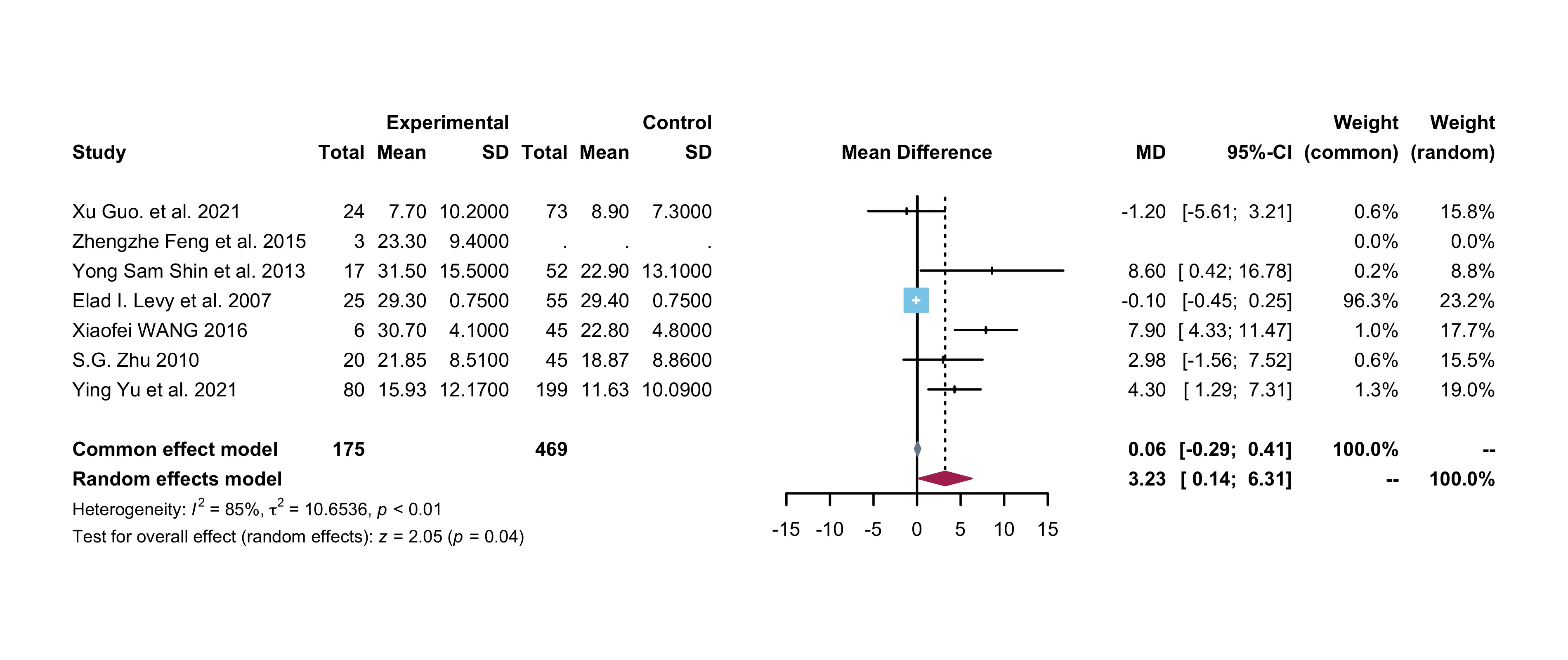

Supplement: Supplementary file 7 [file Image_7.JPEG]

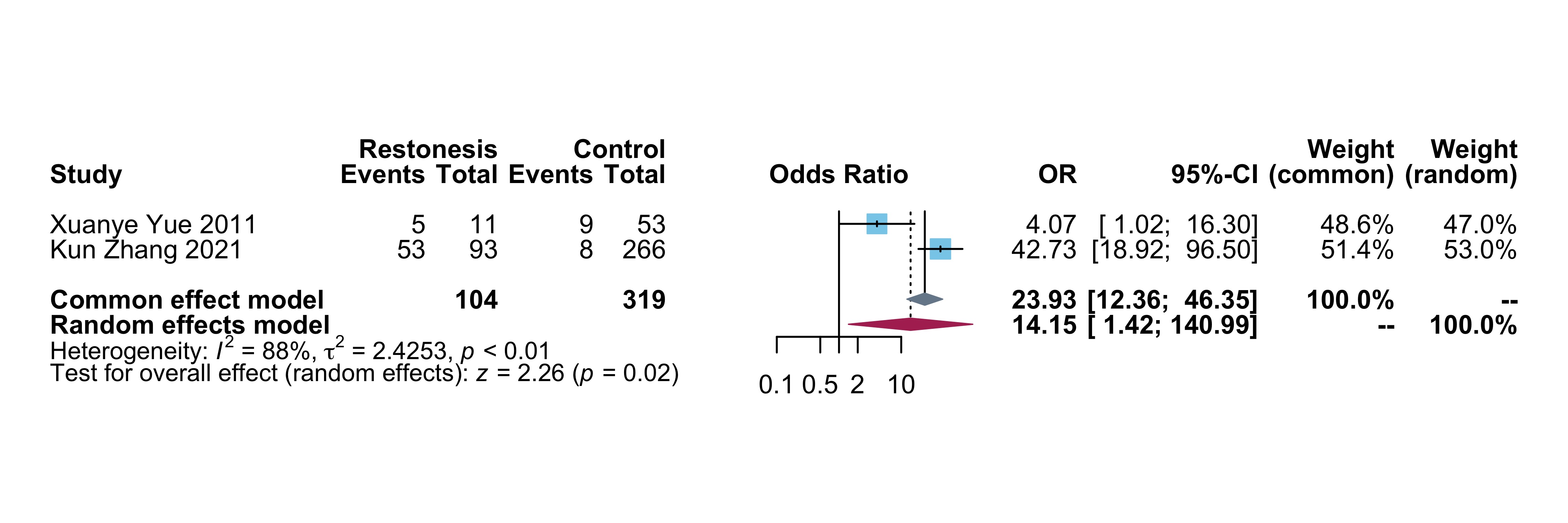

Supplement: Supplementary Figures 5–8 — Possible risk factors for ISR. [file Image_8.JPEG]

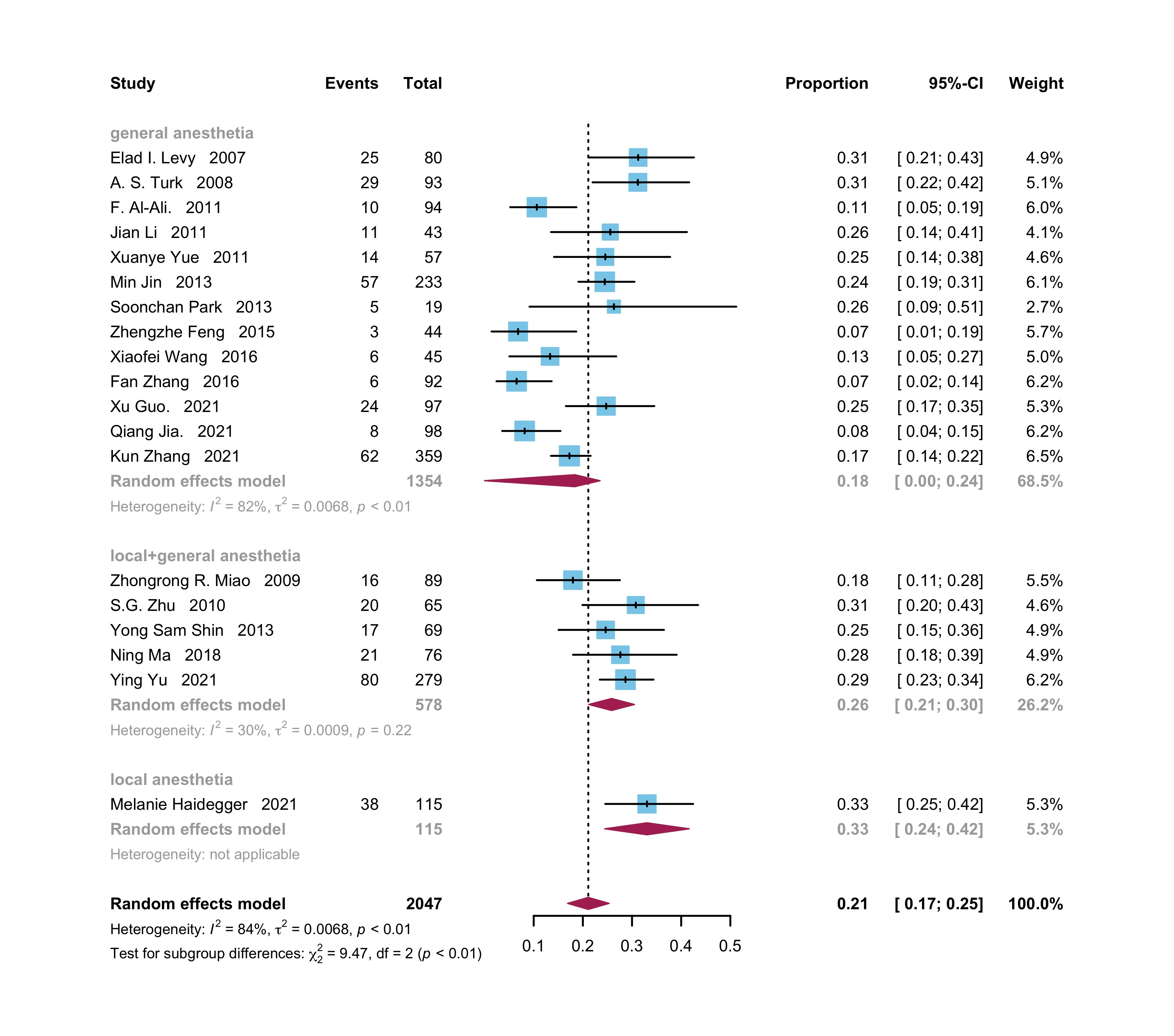

Supplement: Supplementary file 9 [file Image_9.JPEG]

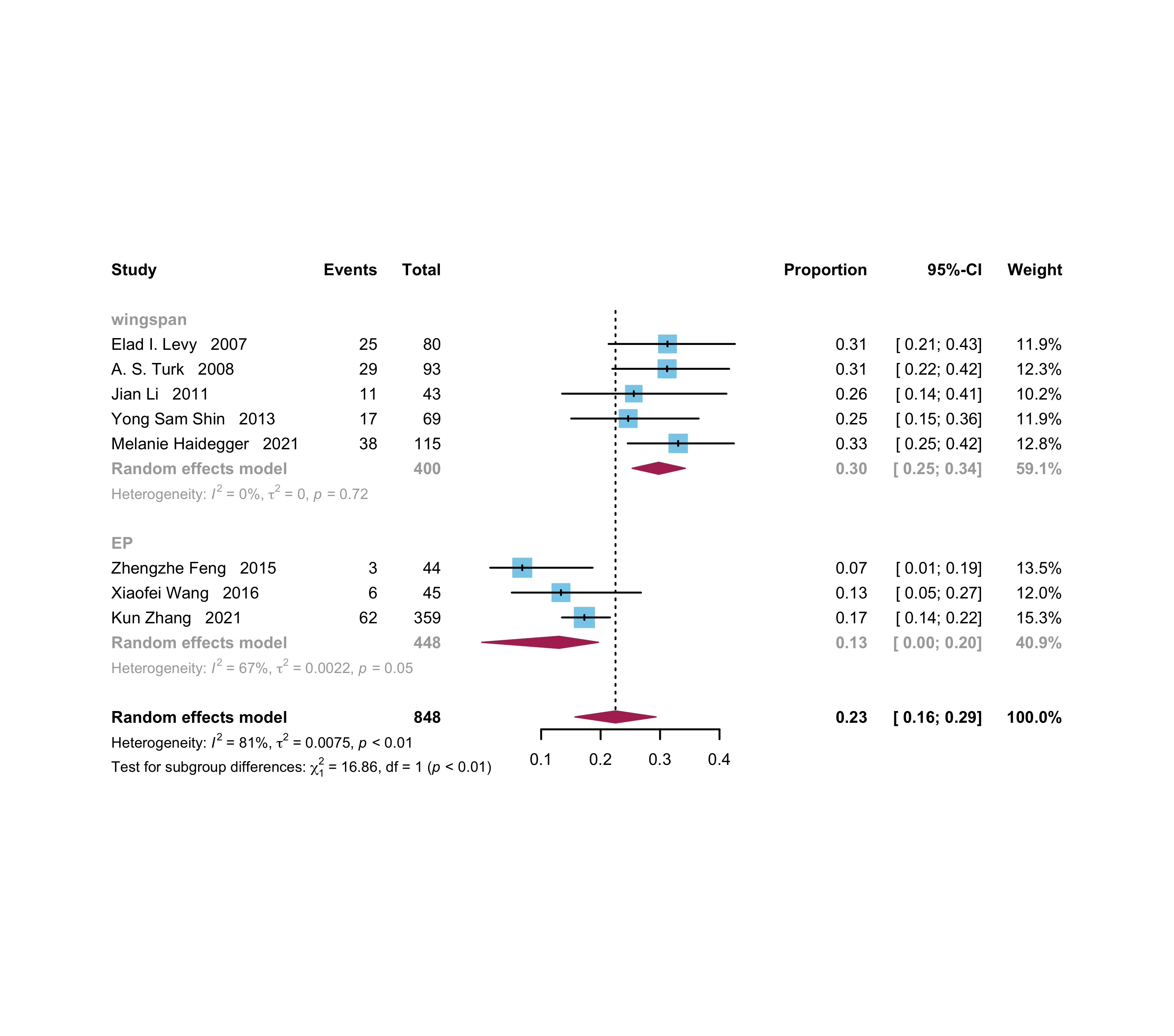

Supplement: Supplementary Figures 9–10 — The result of the subgroup analysis. [file Image_10.JPEG]
