# Supplementary material for: Identifying risk factors for in-stent restenosis in symptomatic intracranial atherosclerotic stenosis: a systematic review and meta-analysis
Source: Front Neurol. 2023 Jul 14;14:1170110. doi: 10.3389/fneur.2023.1170110 (PMC10375724; doi:10.3389/fneur.2023.1170110)
Supplement: Supplementary file 11 [file Table_1.DOCX]

Quality measure of included cohort studies by the Newcastle-Ottawa scale^a^

| Study author | selection^b^ | | | | comparability^c^ | | outcome^d^ | | | overall |
| --- | --- | --- | --- | --- | --- | --- | --- | --- | --- | --- |
|  | 1 | 2 | 3 | 4 | a | b | 1 | 2 | 3 |  |
| F. Al-Ali. Et al.^40^ | ⭐️ | ⭐️ | ⭐️ | ⭐️ | ⭐️ | ⭐️ | ⭐️ | ⭐️ |  | 8 |
| Jian Li et al.^51^ | ⭐️ | ⭐️ | ⭐️ |  | ⭐️ | ⭐️ | ⭐️ | ⭐️ |  | 7 |
| Xuanye Yue et al.^53^ | ⭐️ | ⭐️ | ⭐️ |  | ⭐️ | ⭐️ | ⭐️ | ⭐️ |  | 7 |
| Min Jin et al.^54^ | ⭐️ | ⭐️ | ⭐️ | ⭐️ | ⭐️ | ⭐️ | ⭐️ | ⭐️ |  | 8 |
| Soonchan Park et al.^56^ | ⭐️ | ⭐️ | ⭐️ |  | ⭐️ | ⭐️ | ⭐️ | ⭐️ |  | 7 |
| Fan Zhang et al.^60^ | ⭐️ | ⭐️ | ⭐️ |  | ⭐️ | ⭐️ | ⭐️ | ⭐️ | ⭐️ | 8 |
| Xu Guo et al.^37^ | ⭐️ | ⭐️ | ⭐️ | ⭐️ | ⭐️ | ⭐️ | ⭐️ | ⭐️ |  | 8 |
| Kun Zhang et al.^63^ | ⭐️ | ⭐️ | ⭐️ |  | ⭐️ | ⭐️ | ⭐️ | ⭐️ | ⭐️ | 8 |
| A. S. Turk et al.^48^ | ⭐️ | ⭐️ | ⭐️ |  |  | ⭐️ | ⭐️ | ⭐️ |  | 6 |

^a^ Newcastle-Ottawa Scale for quality assessment for cohort studies (scores 08; studies with >5 was considered high-quality).

^b^ Selection

1) Representativeness of the exposed cohort

a) Truly representative of the average (described) in the community ⭐️

b) Somewhat representative of the average (described) in the community ⭐️

c) Selected group of users, eg, nurses, volunteers

d) No description of the derivation of the cohort

2) Selection of the nonexposed cohort

a) Drawn from the same community as the exposed cohort ⭐️

b) Drawn from a different source

c) No description of the derivation of the nonexposed cohort

3) Ascertainment of exposure

a) Secure record (eg, surgical records) ⭐️

b) Structured interview ⭐️

c) Written self-report

d) No description

4) Demonstration that the outcome of interest was not present at the start of study

a) Yes ⭐️

b) No

^c^ Comparability

1) Comparability of cohorts on the basis of the design or analysis

a) Study controls for (select the most important factor) ⭐️

b) Study controls for any additional factor ⭐️

^d^ Outcome

1) Assessment of outcome

a) Independent blind assessment ⭐️

b) Record linkage ⭐️

c) Self-report

d) No description

2) Was follow-up long enough for outcomes to occur?

a) Yes (select an adequate follow up period for outcome of interest) ⭐️

b) No

3) Adequacy of follow-up of cohorts

a) Complete follow-up; all subjects accounted for ⭐️

b) Subjects lost to follow-up unlikely to introduce bias, small number lost. (select an adequate %) follow-up, or description provided of those lost ⭐️

c) Follow up rate < (select an adequate percentage) and no description of those lost

d) No statement
