# Supplementary material for: Identifying risk factors for in-stent restenosis in symptomatic intracranial atherosclerotic stenosis: a systematic review and meta-analysis
Source: Front Neurol. 2023 Jul 14;14:1170110. doi: 10.3389/fneur.2023.1170110 (PMC10375724; doi:10.3389/fneur.2023.1170110)
Supplement: Supplementary Tables 1, 2 — Detailed quality assessment information was provided. [file Table_2.DOCX]

| MINORS Criteria^a^ | | | | | | | | | |
| --- | --- | --- | --- | --- | --- | --- | --- | --- | --- |
| Study Auther | 1 | 2 | 3 | 4 | 5 | 6 | 7 | 8 | Overall |
| Elad I. Levy et al.^47^ | 2 | 2 | 2 | 2 | 2 | 2 | 2 | 0 | 14 |
| S.G. Zhu et al.^38^ | 2 | 2 | 0 | 2 | 2 | 2 | 0 | 0 | 10 |
| Zhongrong R. Miao et al.^49^ | 2 | 2 | 2 | 2 | 2 | 2 | 0 | 0 | 12 |
| Yong Sam Shin et al.^57^ | 2 | 2 | 2 | 2 | 2 | 2 | 0 | 0 | 12 |
| Zhengzhe Feng et al.^58^ | 2 | 2 | 2 | 2 | 2 | 2 | 2 | 0 | 14 |
| Xiaofei Wang et al.^59^ | 2 | 2 | 2 | 2 | 2 | 2 | 0 | 0 | 12 |
| Ning Ma et al.^61^ | 2 | 2 | 2 | 2 | 2 | 2 | 2 | 0 | 14 |
| Qiang Jia et al.^44^ | 2 | 2 | 0 | 2 | 2 | 2 | 0 | 0 | 10 |
| Melanie Haidegger et al. | 2 | 2 | 2 | 2 | 2 | 2 | 2 | 0 | 14 |
| Ying Yu et al. | 2 | 2 | 2 | 2 | 2 | 2 | 0 | 0 | 12 |

^a^ The MINORS criteria include the following items:

1) A clearly stated aim

2) Inclusion of consecutive patients

3) Prospective data collection

4) End points appropriate for the aim of the study

5) Unbiased assessment of the study end point

6) A follow-up period appropriate for the aims of the study

7) 5% lost to follow-up

8) Prospective calculation of the sample size; additional criteria in the case of a

comparative study

9) An adequate control group

10) Contemporary groups

11) Baseline equivalence of groups

12) Adequate statistical analyses.

The items are scored as follows: 0 (not reported); 1 (reported but inadequate); or

2 (reported and adequate). The ideal global score was 16 for noncomparative studies and 24 for comparative studies. All included single-arm studies were noncomparative studies.
